# Dioxin Receptor Adjusts Liver Regeneration After Acute Toxic Injury and Protects Against Liver Carcinogenesis

**DOI:** 10.1038/s41598-017-10984-w

**Published:** 2017-09-05

**Authors:** Nuria Moreno-Marín, Eva Barrasa, Antonio Morales-Hernández, Beroé Paniagua, Gerardo Blanco-Fernández, Jaime M. Merino, Pedro M. Fernández-Salguero

**Affiliations:** 1 Departamento de Bioquímica y Biología Molecular, Facultad de Ciencias, Universidad de Extremadura, Badajoz, Spain; 2 St. Jude Children’s Research Hospital, Memphis, TN USA; 3 Hospital Universitario Infanta Cristina, Badajoz, Spain

## Abstract

The aryl hydrocarbon receptor (AhR) has roles in cell proliferation, differentiation and organ homeostasis, including the liver. AhR depletion induces undifferentiation and pluripotency in normal and transformed cells. Here, AhR-null mice (*AhR*−/−) were used to explore whether AhR controls liver regeneration and carcinogenesis by restricting the expansion of stem-like cells and the expression of pluripotency genes. Short-term CCl_4_ liver damage was earlier and more efficiently repaired in *AhR*−/− than in *AhR*+/+ mice. Stem-like CK14 + and TBX3 + and pluripotency-expressing OCT4 + and NANOG + cells expanded sooner in *AhR*−/− than in *AhR*+/+ regenerating livers. Stem-like side population cells (SP) isolated from *AhR*−/− livers had increased β-catenin (β-Cat) signaling with overexpression of *Axin2*, *Dkk1* and *Cyclin D1*. Interestingly, *β-Cat*, *Axin2* and *Dkk1* also increased during regeneration but more notably in AhR-null livers. Liver carcinogenesis induced by diethylnitrosamine (DEN) produced large carcinomas in all *AhR*−/− mice but mostly premalignant adenomas in less than half of *AhR*+/+ mice. AhR-null tumoral tissue, but not their surrounding non-tumoral parenchyma, had nuclear β-Cat and Axin2 overexpression. OCT4 and NANOG were nevertheless similarly expressed in *AhR*+/+ and *AhR*−/− lesions. We suggest that AhR may serve to adjust liver repair and to block tumorigenesis by modulating stem-like cells and β-Cat signaling.

## Introduction

Although cell renewal is very scarce in most mammalian tissues under homeostatic conditions, a rapid regenerative response can be observed in certain organs upon exposure to toxic or harmful molecules. Lung and liver are among those organs since they can reestablish normal tissue architecture and functionality from the activation of undifferentiated precursors after treatment with damaging chemicals^1–3^.

Liver regeneration can be experimentally induced by partial hepatectomy or toxic injury, the former exemplified by two-third hepatic resection and the second by acute carbon tetrachloride (CCl_4_) treatment^2–4^. The mechanisms and cell types involved differ between both processes. While partial hepatectomy predominantly activates the division of quiescent hepatocytes^3, 4^, the response to acute toxic damage appears to be associated to the proliferation of hepatocytes and to a plausible increase in undifferentiated liver stem-like cells^4–7^. Hepatic CCl_4_ toxicity involves necrotic cell death and an acute inflammatory reaction caused by the infiltration of polymorphonuclear leukocytes and macrophages to the dead tissue^2^. Undifferentiated cells expressing pluripotency-inducing factors also appear to have a significant role in liver cancer progression and dissemination^8–10^. Despite some controversy about the phenotype of liver stem cells, certain molecular markers including cytokeratin 14 (CK14)^11, 12^ and TBX3^13^ have been proposed to define early progenitor liver cells. Interestingly, a subpopulation of cells expressing Axin2 and TBX3 has been identified close to the central vein whose self-renewing potential is dependent on Wnt signals produced by the endothelium^13^. In addition, recent studies have identified pluripotency and reprogramming factors OCT4, NANOG and SOX2 as relevant molecular intermediates able to sustain stem-like properties in the liver and the progression of hepatocellular carcinomas from hepatic cancer stem cells^14–18^.

The aryl hydrocarbon/dioxin receptor (AhR) interacts with signalling pathways controlling not only the cell response to toxic and carcinogenic compounds but also relevant physiological functions^19, 20^. Recent work suggests that AhR may have a causal role in maintaining cell differentiation in the immune system^21–24^, gonads^25^, melanoma tumors^26, 27^ and skin epithelium^28^. AhR expression appears also relevant in embryonic stem cells^29, 30^, osteoblasts^31, 32^ and embryonic teratocarcinoma cells^33^. Notably, AhR deficiency exerts a profound impact in the hepatic system so that AhR-null mice have reduced liver size and portal fibrosis^34–36^ and a persistent intrahepatic porto-systemic shunt^37, 38^.

Previous studies have shown that AhR activation by its prototype carcinogenic ligand TCDD (2,3,7,8-tetrachlorodibenzo-p-dioxin) inhibits caudal fin and heart regeneration in zebrafish larvae, suggesting a link between AhR signalling and activation of embryonic-like cells early in development^39^. In mammals, few reports have shown that TCDD can partially impair liver regeneration in mice after two-third partial hepatectomy possibly by controlling the levels of cyclin-dependent kinase inhibitors p21^Cip1^ and p27^Kip1^ ^40, 41^.

In this work, we have used AhR wild type (*AhR*+/+) and AhR-null (*AhR*−/−) mice to investigate how AhR deficiency affects the activation of stem-like and pluripotent cells in the liver during regeneration after acute exposure to the hepatotoxin CCl_4_ and in hepatic tumors induced by the carcinogen diethylnitrosamine (DEN). We have found that AhR depletion improved liver regeneration and promoted the growth of large hepatocarcinomas likely by a mechanism involving increased proliferation and the activation of cells expressing stemness markers. These results could be of interest to develop non-toxic AhR antagonists to restore liver structure and function after toxic injury or after surgical intervention. Non-toxic AhR agonists could be also useful for inhibiting liver tumor growth. Consequently, lack of AhR could be also considered a bad prognostic value in liver cancer.

## Results

### AhR deficiency improves liver regeneration after acute toxic injury

**Figure 1:**
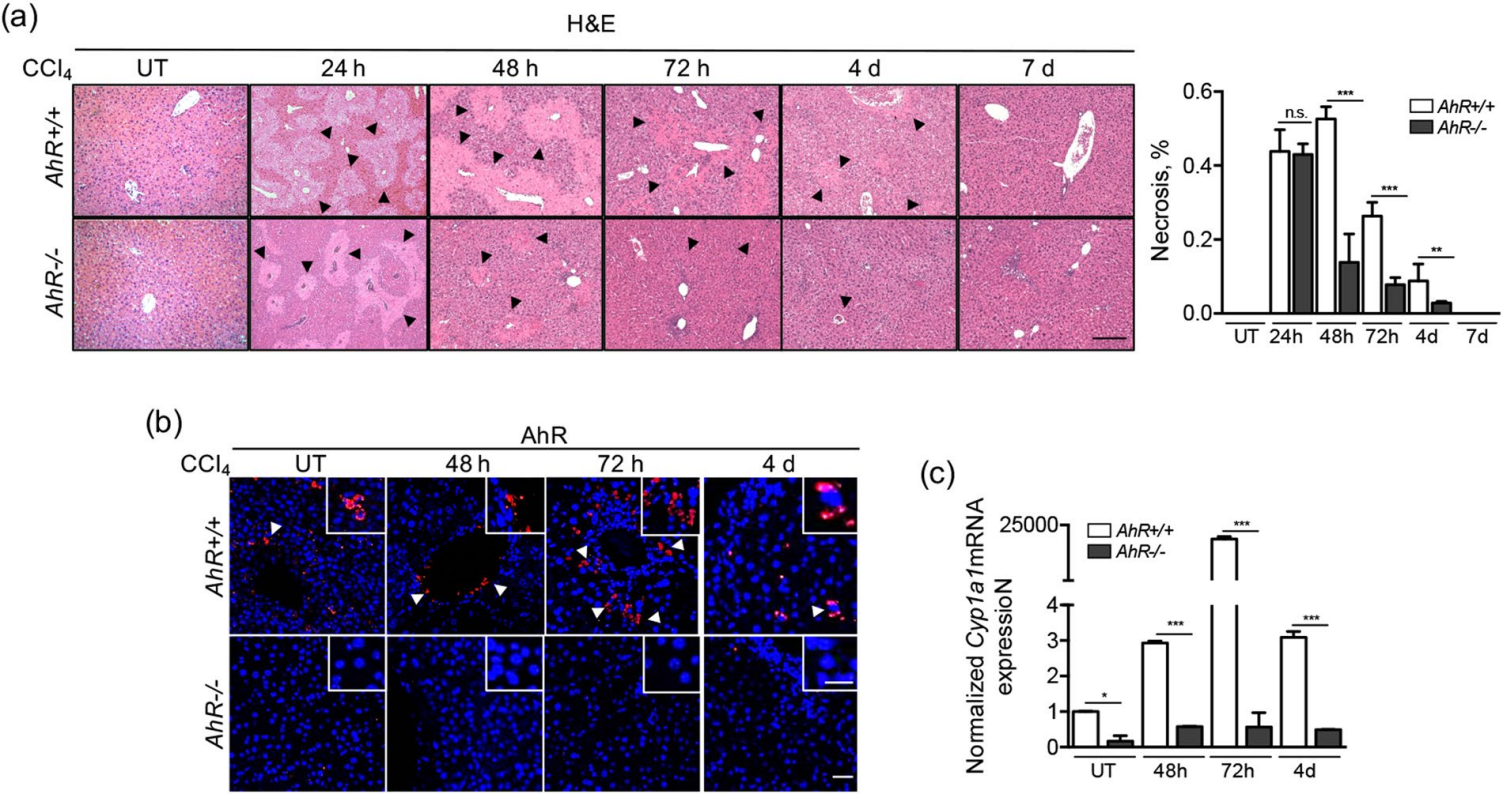
AhR deficiency improves recovery from CCl_4_ induced necrosis. *AhR*+/+ and *AhR*−/− male mice were left untreated (UT) or treated with 0.7 ml/Kg CCl_4_ and liver regeneration determined at 24 h, 48 h, 72 h, 4 days and 7 days. **(a)** Liver sections were processed and stained by histological analyses using hematoxylin-eosin (H&E). Necrotic areas were quantified blinded by 6 independent observers (right panel)**. (b)** AhR expression was analyzed by immunofluorescence using a specific antibody. An Alexa 633-conjugated secondary antibody was used for detection. DAPI staining was used to label cell nuclei. **(c)** The expression of the AhR canonical target gene *Cyp1a1* was determined by RT-qPCR in the regenerating liver using total liver RNA and the oligonucleotides indicated in Supplementary Table 1. Gene expression was normalized by *Gapdh* and represented as 2^−ΔΔCt^. Four to five *AhR*+/+ and *AhR*−/− mice were used at each time point and at least 3 technical replicates per mouse were performed. A NIKON TE2000U microscope was used for immunohistochemistry. An Olympus FV1000 confocal microscope and the FV10 software (Olympus) were used for immunofluorescence. Bars: panel (a) 100 μm, panel (b) 50 μm; inset 25 μm. Data are shown as mean ± SD. *p < 0.05, **p < 0.01, ***p < 0.001, n.s. not statistically significant. Arroheads mark necrotic areas in the liver parenchyma (panel a) or AhR positive cells (panel b).

We have used the liver response to acute CCl_4_ toxicity as an experimental approach to analyze the role of AhR in tissue regeneration. A single dose of CCl_4_ induced severe organ damage with ample necrotic areas surrounding the central vein and periportal areas of the hepatic parenchyma in both *AhR*+/+ and *AhR*−/− mice. (Fig. 1a). However, quantification of necrotic regions revealed that regeneration took place faster and more efficiently in *AhR*−/− mice since very few necrotic lesions remained at 72 h from treatment and their parenchyma was fully restored by 4 days. *AhR*+/+ mice, on the contrary, exhibited a delayed regenerative process that became significant at 72 h, persisting their hepatic lesions for at least 4 days (Fig. 1a). Nonetheless, regeneration was completed by 7 days from CCl_4_ administration in both genotypes. AhR expression transiently increased during the repair process in CCl_4_ exposed *AhR*+/+ mice, reaching a maximum at 72 h in cells located close to the central vein to decrease to very low levels at 4 days (Fig. 1b). AhR induced by the regenerative process was transcriptionally active as determined by the marked induction of its canonical target gene *Cyp1a1*, that reached maximum levels at 72 h **(**Fig. 1c **)**. As expected, AhR expression or *Cyp1a1* induction were undetectable in *AhR*−/− livers (Fig. 1b).

**Figure 2:**
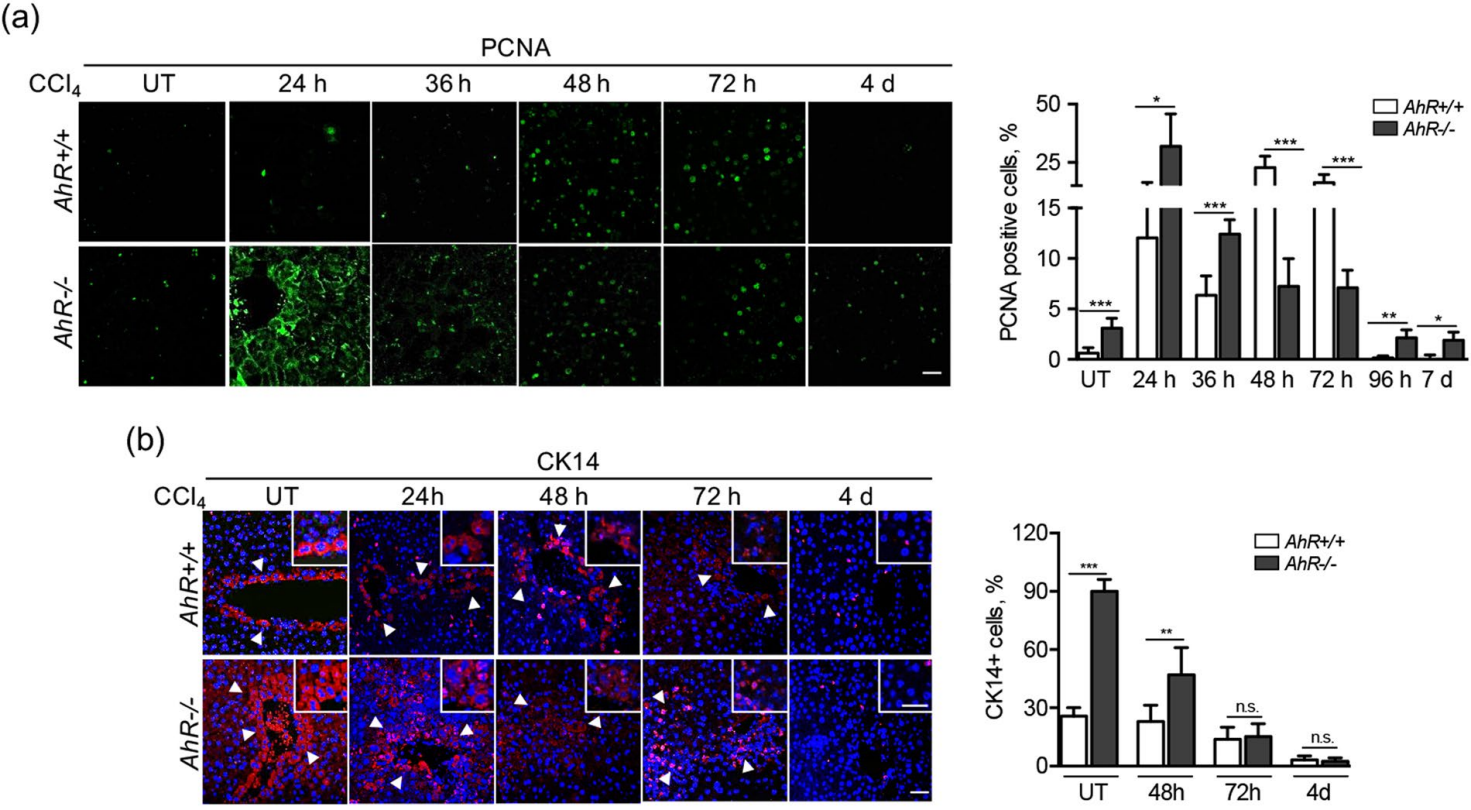
AhR-null livers develop an early proliferative response to CCl_4_ treatment. *AhR*+/+ and *AhR*−/− male mice were left untreated (UT) or treated with 0.7 ml/Kg CCl_4_ and liver regeneration determined at 24 h, 48 h, 72 h, 4 days and 7 days. **(a)** Liver sections were analyzed for the presence of PCNA positive cells by immunofluorescence. The time-dependent kinetic was quantified for each time point and the results are shown in the right panel. **(b)** The presence of cells expressing the stem-like marker CK14 was analyzed by immunofluorescence in liver sections using a specific antibody. An Alexa 633-conjugated secondary antibody was used for detection. DAPI staining was used to label cell nuclei. Quantification of the results is shown in the right panel. Four to five *AhR*+/+ and *AhR*−/− mice were used at each time point and at least 3 technical replicates per mouse were performed. A NIKON TE2000U microscope was used for immunohistochemistry. An Olympus FV1000 confocal microscope and the FV10 software (Olympus) were used for immunofluorescence. Bars: panel (a) 50 μm, panel (b) 100 μm; inset 25 μm. Data are shown as mean ± SD. *p < 0.05, **p < 0.01, ***p < 0.001, n.s. not statistically significant. Arroheads mark CK14 positive cells (panel b).

Liver regeneration after CCl_4_ treatment involved enhanced cell proliferation in *AhR*+/+ and *AhR*−/− mice, although the kinetic with time differed between both genotypes. Proliferating cell nuclear antigen (PCNA) expression rapidly increased in *AhR*−/− livers reaching maximum values at 24 to 36 h to gradually decrease at later time points **(**Fig. 2a **)**. PCNA positive cells also accumulated in *AhR*+/+ livers although their kinetic was delayed until 48 to 72 h from CCl_4_ exposure **(**Fig. 2a **)**. Interestingly, *AhR*−/− but not *AhR*+/+ livers had a significant amount of cytosolic PCNA protein at early stages of regeneration, probably resulting for a nuclear to cytoplasmic shuttling process that takes place to protect from apoptosis and to sustain cell survival and resistance to toxic agents^42, 43^. Thus, the more efficient regeneration observed in AhR-null livers involved an earlier proliferative response.

### AhR modulates the generation of cell types expressing stem-like and pluripotency markers after CCl4 induced toxicity

**Figure 3:**
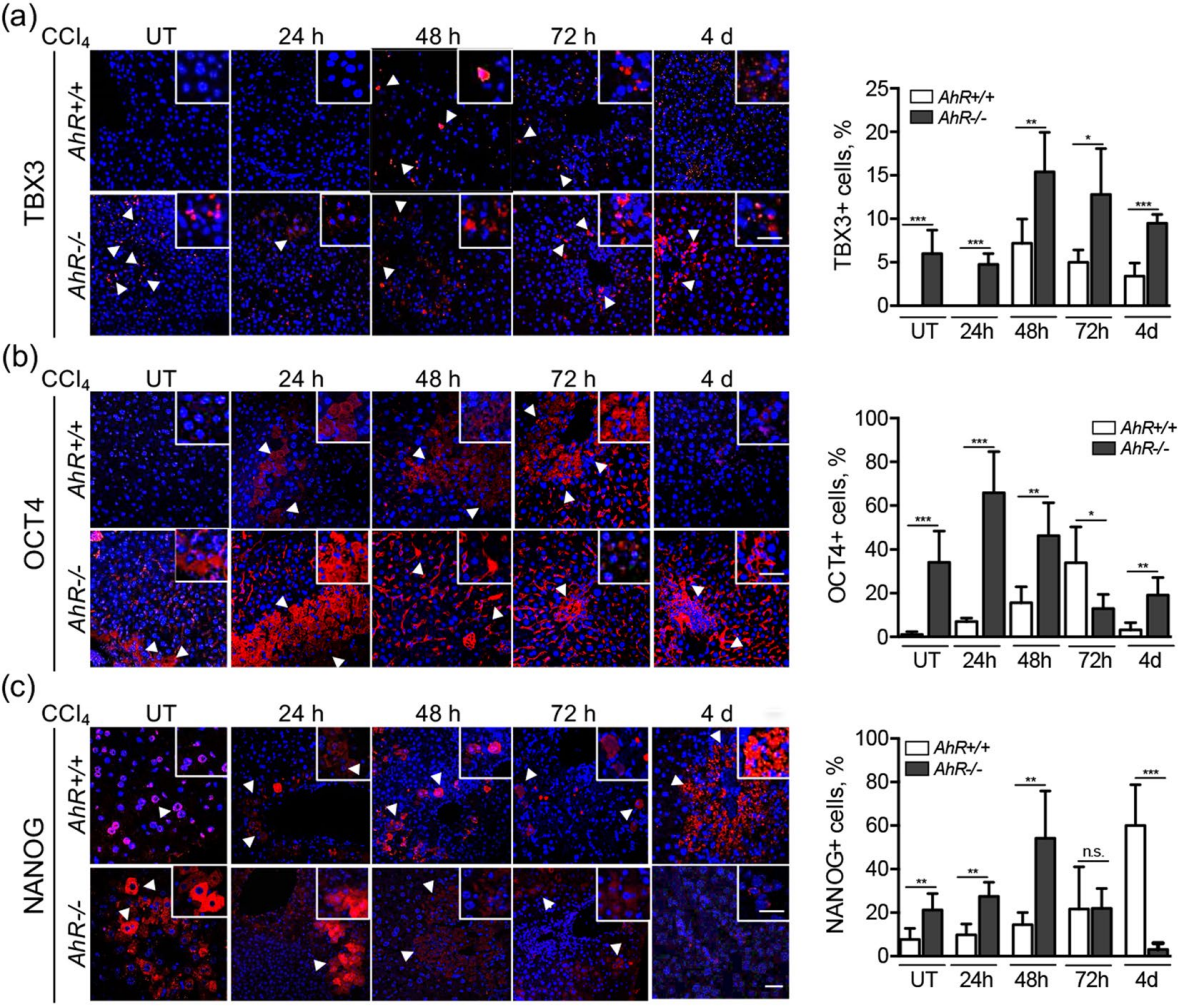
Increased liver progenitors and upregulated pluripotency genes could contribute to liver regeneration in *AhR*−/− mice. *AhR*+/+ and *AhR*−/− male mice were left untreated (UT) or treated with 0.7 ml/Kg CCl_4_ and liver regeneration determined at 24 h, 48 h, 72 h and 4 days. TBX3 **(a)**, OCT4 **(b)** and NANOG **(c)** positive cells were detected by immunofluorescence in liver sections using specific antibodies. An Alexa 633-conjugated secondary antibody was used for detection. DAPI staining was used to label cell nuclei. Data for each marker were quantified and the results are shown in the right panels. Four to five *AhR*+/+ and *AhR*−/− mice were used at each time point and at least 3 technical replicates per mouse were performed. An Olympus FV1000 confocal microscope and the FV10 software (Olympus) were used for immunofluorescence. Bar: 50 μm; inset 25 μm. Data are shown as mean ± SD. *p < 0.05, **p < 0.01, ***p < 0.001, n.s. not statistically significant. Arroheads mark TBX3 positive (panel a), OCT4 positive (panel b) or NANOG positive (panel c) cells.

Liver regeneration after exposure to toxic chemicals such as CCl_4_ seems to predominantly involve the activation of stem-like cells expressing, among others, CK14, c-kit and Flt3^11, 12, 44^ markers. Resident primary hepatocytes could also contribute to resolve necrotic tissue damage induced by CCl_4_^3, 45^. *AhR*−/− livers had increased basal numbers of CK14-expressing cells as compared to *AhR*+/+ mice (Fig. 2b). Shortly after CCl_4_ treatment (e.g. 48 h), CK14 expression was maintained in *AhR*+/+ livers and moderately decreased in *AhR*−/− livers. At later times from 72 h to 4 days, CK14 decreased to very low levels in both genotypes (Fig. 2b). T-box transcription factor TBX3 is considered an early marker of pericentral liver progenitor cells^13^. Untreated *AhR*−/− livers had detectable numbers of TBX3 positive cells that markedly increased at 48 and 72 h after CCl_4_ treatment. Basal *AhR*+/+ livers did not show detectable TBX3 positivity and this cell type only became induced at 48 h following CCl_4_ administration **(**Fig. 3a **)**. We have previously reported that AhR represses pluripotency factors OCT4 and NANOG in human embryonic teratocarcinoma cells^33^. OCT4 expressing cells were significantly more abundant in basal *AhR*−/− liver parenchyma than in *AhR*+/+ livers **(**Fig. 3b **)**. After CCl_4_ treatment, OCT4 expression peaked from 24 to 48 h in AhR-null mice to then progressively decrease at 72 h and 4 days (Fig. 3b). In *AhR*+/+ livers, OCT4 positive cells also increased during regeneration but with a delayed kinetic reaching its maximum at 72 h and decaying to initial levels at 4 days (Fig. 3b). Endogenous expression of NANOG was also higher in *AhR*−/− livers and became maximally induced at 48 h **(**Fig. 3c **)**. Similarly to OCT4, NANOG positive cells were less abundant in naive *AhR*+/+ livers and they did not significantly accumulate until late regeneration at 4 days (Fig. 3c).

### *AhR−/−* livers have increased number of side population cells and upregulation of the β-Cat pathway

**Figure 4:**
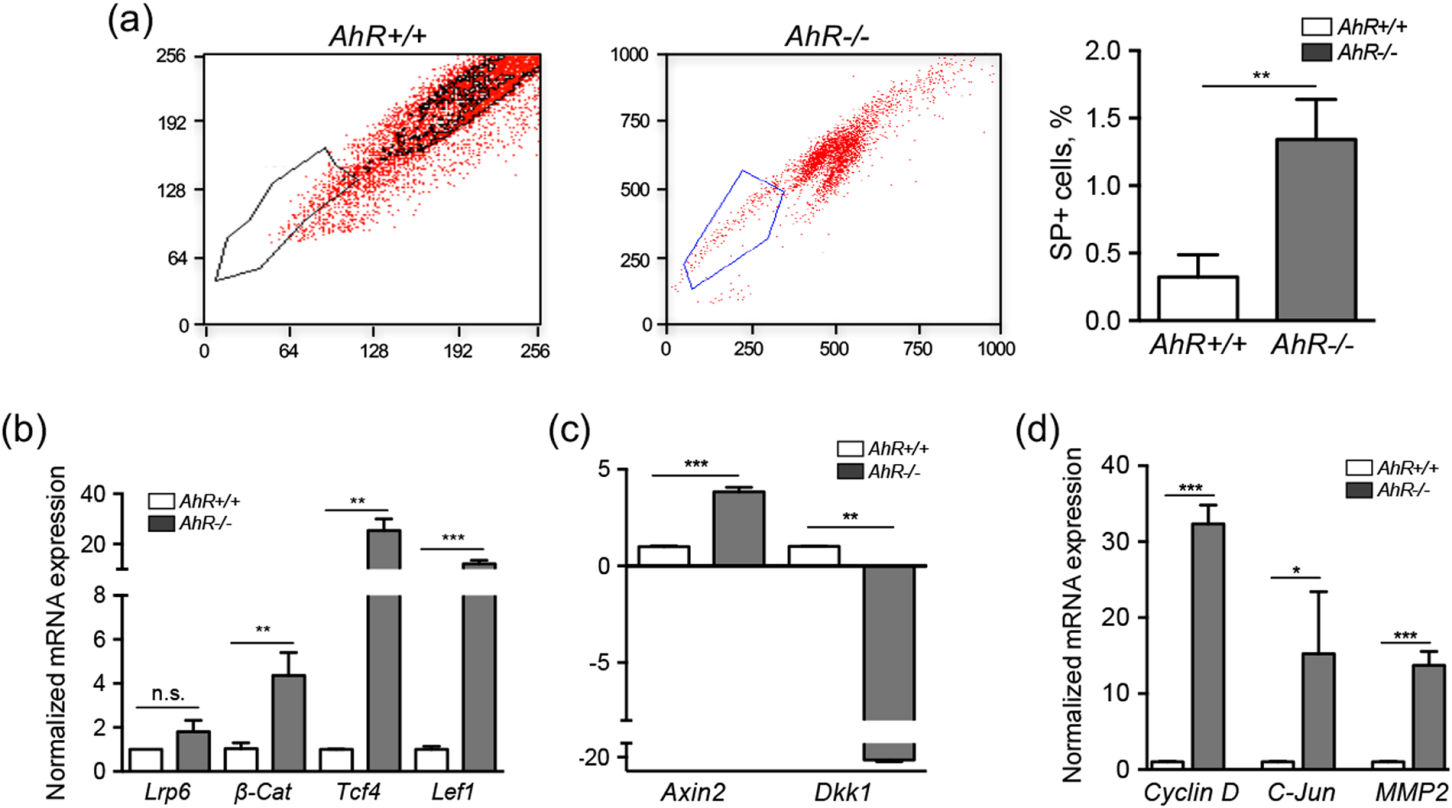
*AhR*−/− livers have incrased levels of SP cells and upregulation of the Wnt/β-Cat signaling pathway. (**a**) Livers were obtained from *AhR*+/+ and *AhR*−/− mice and processed to isolate and quantify side population cells (SP) by cell sorting. SP + cells are expressed with respect to the total number of liver cells. **(b)** mRNA was purified from SP cells isolated from both genotypes and used to analyze by RT-qPCR the mRNA expression of *β-Cat* and of molecular intermediates of the Wnt/β-Cat pathway *Lrp6*, *Tcf4* and *Lef1*. **(c,d)** mRNA expression of Wnt/β-Cat canonical target genes *Axin2* and *Dkk1* **(c)** and *Cyclin D1*, *c-Jun* and *Mmp2* **(d)** was also analyzed by RT-qPCR. Oligonucleotides used are indicated in Supplementary Table 1. Gene expression was normalized by *Gapdh* and represented as 2^−ΔΔCt^. Four *AhR*+/+ and *AhR*−/− mice were used and 3 experimental replicates following SP isolation were analyzed. Data are shown as mean ± SD. *p < 0.05, **p < 0.01, ***p < 0.001, n.s. not statistically significant.

**Figure 5:**
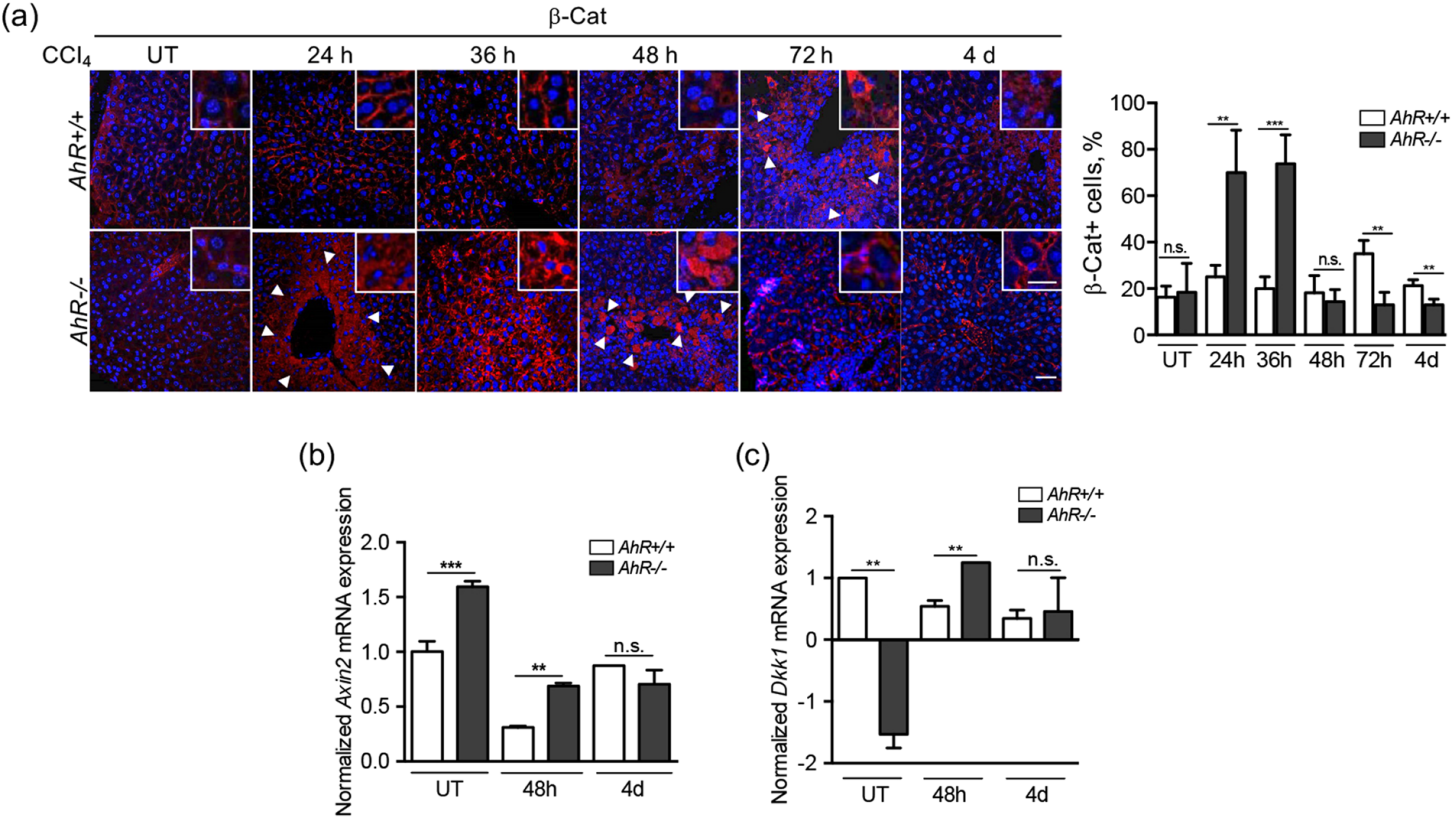
β-Cat signaling is activated during liver regeneration in an AhR-dependent manner. *AhR*+/+ and *AhR*−/− male mice were left untreated (UT) or treated with 0.7 ml/Kg CCl_4_ for the indicated times. **(a)** Livers were obtained, fixed in formalin, embedded in paraffin and processed for β-Cat detection by immunofluorescence. Quantification of the results is shown in the right panel. **(b,c)** mRNA expression of *Axin2* **(b)** and *Dkk1* **(c)** was analyzed by RT-qPCR in liver samples from *AhR*+/+ and *AhR*−/− mice at the indicated times after CCl_4_ treatment. Oligonucleotides used are indicated in Supplementary Table 1. Gene expression was normalized by *Gapdh* and represented as 2^−ΔΔCt^. Four to five *AhR*+/+ and *AhR*−/− mice were used at each time point and at least 3 technical replicates per mouse were performed. Bar: 50 μm; inset 25 μm. Data are shown as mean ± SD. **p < 0.01, ***p < 0.001, n.s. not statistically significant. Arroheads mark β-Cat positive cells (panel a).

We next used cell sorting to analyze the stem-like side population (SP) in untreated *AhR*+/+ and *AhR*−/− mouse livers. Complete livers from naive *AhR*+/+ and *AhR*−/− mice were processed, stained for the stem-like markers CD133+ , CD44+ and CD29+ and positive cells isolated and characterized (see the Methods). AhR deficient livers had a significant increase in SP cells with respect to wild type livers **(**Fig. 4a **)**, which is consistent with their enhanced content in cell populations expressing progenitor and pluripotency markers. Recent studies have associated the stem cell phenotype in the liver with the upregulation of the Wnt/β-Cat signaling pathway and the activation of pluripotency factors OCT4, NANOG, TBX3 and SOX2^46–48^. We therefore decided to analyze the Wnt/β-Cat pathway in SP cells isolated from *AhR*+/+ and *AhR*−*/*− livers. mRNA expression of *β-Cat* and of its transcriptional partners *Tcf4* and *Lef1* was markedly increased in *AhR*−/− SP cells as compared to *AhR*+/+ SP cells **(**Fig. 4b **)**. No significant differences were observed for the Wnt receptor *Lrp6* between both genotypes **(**Fig. 4b **)**. Consistently, the expression of the canonical Wnt/β-Cat target gene *Axin2* was increased whereas that of Dickkopf-related protein 1 (*Dkk1*) was markedly repressed in *AhR*−/− SP cells **(**Fig. 4c **)**. Additional Wnt/β-Cat target genes *Cyclin D1, c-Jun* and matrix metalloproteinase-2 (*Mmp2*) were also overexpressed in *AhR*−/− SP cells with respect to *AhR*+/+ SP cells **(**Fig. 4d **)**. The analysis of liver sections revealed similar levels of β-Cat protein in untreated *AhR*+/+ and *AhR*−/− mice **(**Fig. 5a **)**. However, early during regeneration (24 h), β-Cat significantly accumulated in the pericentral region of *AhR*−/− livers and such accumulation persisted for at least 36 h, to then return to basal levels at 48 h **(**Fig. 5a **)**. *AhR*+/+ livers, on the contrary, only showed moderate β-Cat localization to pericentral areas at 72 h from CCl_4_ treatment **(**Fig. 5a **)**. *Axin2* mRNA was overexpressed under basal conditions in *AhR*−/− livers and its levels decreased from 48 h in parallel with the reduction in β-Cat protein **(**Fig. 5b **)**. In *AhR*+/+ livers, *Axin2* mRNA also decreased at 48 h from CCl_4_ exposure to return to basal levels at later times **(**Fig. 5b **)**. Basal *Dkk1* mRNA expression was repressed in *AhR*−/− livers as compared to *AhR*+/+ livers, but transiently increased at 48 h from treatment. In AhR expressing livers, *Dkk1* became steadily repressed along the regenerative process **(**Fig. 5c **)**. Thus, β-Cat signaling was enhanced in stem-like SP liver cells and at early stages of liver regeneration *in vivo*.

### AhR deficiency increases liver tumor progression by diethylnitrosamine (DEN)

**Figure 6:**
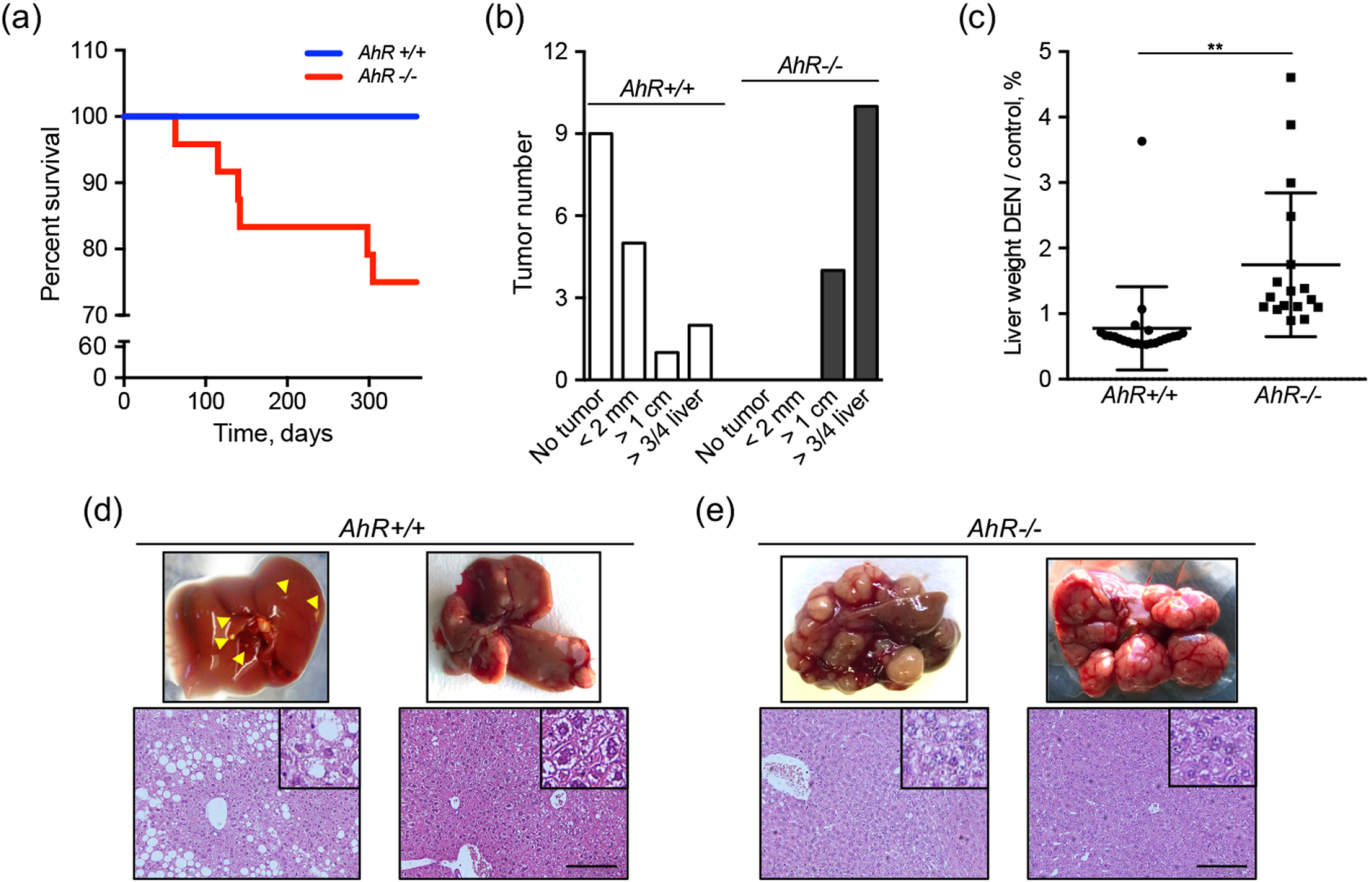
Lack of AhR increases the response to DEN-induced liver tumors. *AhR*+/+ and *AhR*−/− male mice were treated with the carcinogen DEN twice at 4 and 5 weeks of age. **(a)** Kaplan-Meier survival curve for a 1 year period since treatment. Surviving mice were killed at the end of the experiment. **(b)** Liver tumors were recovered from mice and classified in three ranges of sizes corresponding to <2 mm^3^, >1 cm^3^ and those of more than ¾ of the liver. **(c)** Livers from DEN-treated *AhR*+/+ and *AhR*−/− mice were weighted and the results represented as percentage with respect to the average liver weight of untreated wild type and AhR-null mice. **(d)** Representative mild lesion (left) and grown liver tumor (right) from DEN-treated *AhR*+/+ mice are shown. Hematoxylin-eosin stained sections from the corresponding tumors are shown below. Arrowheads indicate liver lesions in *AhR*+/+ livers. **(e)** Representative fully-grown liver carcinomas from DEN-treated *AhR*−/− mice. Hematoxylin-eosin stained sections from the corresponding tumors are shown below. A total of 30 *AhR*+/+ and 30 *AhR*−/− mice were used. A set of 10 control mice for each genotype were also analyzed. Data are shown as mean ± SD. **p < 0.01. Bar: 100 μm.

We next used DEN-induced liver tumors to investigate if tumors growing in AhR deficient mice have altered expression of AhR-regulated pluripotency genes and changes in β-Cat activation. Acute DEN treatment at young age (e.g. 4 and 5 weeks after birth) did not affect survival of *AhR*+/+ mice during a 1 year period. However, it did significantly affect the viabilility of *AhR*−/− mice since close to 25% of the animals died between 4 and 12 months after treatment **(**Fig. 6a **)**. Mice surviving 12 months after DEN treatment were killed and their tumors used for analyses. In agreement with a previous report^49^, AhR deficiency induced massive liver tumors larger than 1 cm^3^ in 25% of the animals; tumors invaded more than three quarters of the liver in the remaining 75% of *AhR*−/− mice **(**Fig. 6b **)**. Almost half of *AhR*+/+ mice did not develop any distinguishable liver tumor and only a small fraction had lesions invading large areas of the liver or with sizes over 1 cm^3^ **(**Fig. 6b **)**. In fact, tumor growth significantly increased total liver weight in AhR-null but not in wild type mice **(**Fig. 6c **)**. Macroscopic evaluation identified small lesions (Fig. 6d, upper left) with steatosis in most *AhR*+/+ mice despite some animals having larger size tumors (Fig. 6d, upper right). *AhR*−/− mice, on the contrary, developed tumors with marked multiplicity and extensive vascularization and lymphocyte infiltration (Fig. 6e, upper). Pathological examination revealed that even the larger *AhR*+/+ tumors were pre-malignant adenomas whereas a significant fraction of those present in *AhR*−/− mice progressed to malignant hepatocarcinomas **(**Fig. 6d,e **)**.

**Figure 7:**
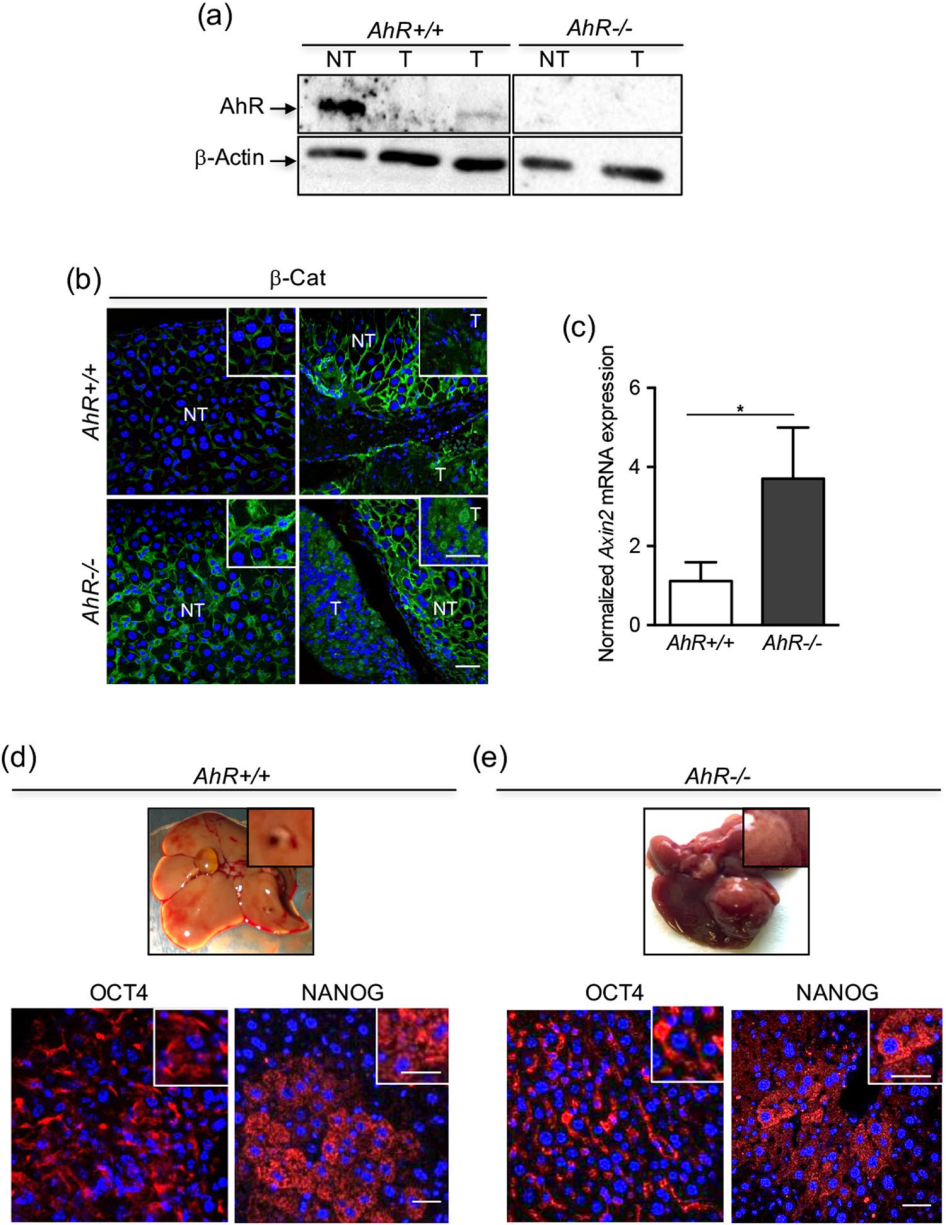
β-Cat is overactivated in *AhR*−/− liver tumors but OCT4 and NANOG are not significantly altered. *AhR*+/+ and *AhR*−/− male mice were treated with the carcinogen DEN twice at 4 and 5 weeks of age. **(a)** AhR protein expression was determined by immunoblotting in the tumor proper (T) and in surrounding non-tumoral tissue (NT) from *AhR*+/+ and *AhR*−/− mice. β-Actin was used to normalize protein levels. *AhR*+/+ and *AhR*−/− samples correspond to non-adjacent lanes from the same blot. **(b)** β-Cat protein was analyzed by immunofluorescence in *AhR*+/+ and *AhR*−/− tumoral tissue (T) and in surrounding non-tumoral stroma (NT). **(c)** *Axin2* mRNA levels were quantified by RT-qPCR in tumors obtained from livers of both genotypes. **(d,e)** Protein expression of OCT4 and NANOG were analyzed by immunofluorescence in *AhR*+/+ and *AhR*−/− liver tumors. Oligonucleotides used are indicated in Supplementary Table 1. Gene expression was normalized by *Gapdh* and represented as 2^−ΔΔCt^. Four biological replicates and 3 experimental replicates per mouse were analyzed. Bar: 50 μm; inset 25 μm. Data are shown as mean ± SD. *p < 0.05.

Protein analysis of tumors growing in *AhR*+/+ mice revealed a marked downregulation of AhR expression in the tumor proper (T) as compared to surrounding non-tumoral tissue (NT) from the same livers. AhR was undetectable in tumoral (T) and non-tumoral tissue (NT) of *AhR*−/− mice **(**Fig. 7a **)**. Thus, liver tumor progression may be associated to AhR downregulation. β-Cat protein was barely detectable in non-tumoral tissue (NT) of *AhR*+/+ livers but highly expressed in surrounding non-tumoral tissue (NT) of *AhR*−/− livers (Fig. 7b, left panels). β-Cat was moderately expressed in largest *AhR*+/+ tumors (T) (Fig. 7b, upper right) but highly expressed and with nuclear localization in most *AhR*−/− tumors (T) (Fig. 7b, lower right). Consistently, mRNA expression of the β-Cat target gene *Axin2* was significantly higher in *AhR*−/− than in *AhR*+/+ liver tumors **(**Fig. 7c **)**. We next used immunofluorescence to determine whether liver tumor growth involved changes in the expression of OCT4 and NANOG. We found that both OCT4 and NANOG were similarly expressed and with a comparable cellular distribution in *AhR*+/+ and *AhR*−/− liver tumors, with a more punctuate pattern in the case of NANOG **(**Fig. 7d,e **)**.

## Discussion

With the exception of the lung in mammals and the caudal fin in zebrafish, tissue regeneration is a rare event and mostly restricted to the liver. Mammalian liver can be induced to regenerate to its initial mass after exposure to necrotic inducing agents or after tissue loss caused by partial hepatectomy^2, 3^. Although some controversy exists, it is generally accepted that liver regeneration after partial hepatectomy is largely hepatocyte-dependent whereas regeneration in response to toxic compounds such as CCl_4_ relies in liver progenitor stem-like cells with some contribution from primary hepatocytes^2–4^. To date, little is known about the mechanisms and molecular intermediates that initiate, limit and terminate liver regeneration. Previous work has shown that AhR activation by TCDD can block heart and caudal fin regeneration in zebrafish^50, 51^. In the mouse, TCDD inhibited liver regeneration after two-third partial hepatectomy possibly by blocking the cell cycle through a p21^Cip1^-dependent mechanism^40, 41^. A recent study has revealed that TCDD enhanced the toxic effects of CCl_4_ in mouse liver probably by increasing cytochrome P450-2E1 expression in an AhR-dependent manner^52^. Here, we have investigated liver regeneration after CCl_4_-induced damage and the liver response to the tumor inducing agent DEN in AhR-null background. The main conclusion from this work is that AhR deficiency improves liver regeneration after acute toxic damage but promotes hepatocarcinoma development likely by the expansion of stem-like cells and the upregulation of pluripotency-related factors including β-Cat. From a clinical point of view, this work indicates that AhR deficiency could be beneficial to increase liver regeneration after toxic injury or tissue loss (e.g. from partial hepatectomy) but detrimental as a potential cause of liver carcinogenesis.

AhR depletion improved liver regeneration after CCl_4_-induced injury including a more efficient and complete repair of necrotic regions surrounding the central vein and periportal areas, despite *AhR*−/− mice having similar sensitivity to this toxic compound. The more competent regeneration observed in *AhR*−/− livers was probably the consequence of an earlier proliferative response reaching maximum values by 24–36 h after injury, whereas it took over 48 h in *AhR*+/+ livers, in agreement with previous data from transplanted wild type mice^53, 54^. Consistently with current hypotheses^2, 3^, the accelerated repair in AhR-lacking mice could be related to the activation of progenitor stem-like cells residing in the liver under basal conditions. Accordingly, cells expressing adult liver progenitor markers CK14 and TBX3^11–13^ were more abundant in *AhR*−/− livers and, importantly, they expanded soon at the beginning of the regeneration process, closely following the kinetics of proliferation and repair. Pluripotency factors OCT4^33, 55, 56^ and NANOG^33^, both regulated by AhR in human embryonic teratocarcinoma cells, were also overexpressed basally and shortly after liver repair in AhR-null mice. These data suggest that, in absence of AhR, enlarged pools of liver cells with stem-like characteristics may trigger an earlier regenerative reaction ultimately resulting in a more efficient repair. Therefore, AhR could serve as a limiting factor controlling physiological regeneration in order to avoid liver overgrowth. Such possibility agrees with the fact that AhR becomes upregulated in wild type livers around the time window when repair was about to be completed.

Enhanced Wnt/β-Cat signaling has been involved in the proliferation of normal and transformed liver cells, at least in part due to the upregulation of a set of pluripotency factors including OCT4 and NANOG^15–18, 48^. Interestingly, stem-like side population cells isolated from *AhR*−/− liver under physiological conditions showed upregulation of canonical Wnt/β-Cat signaling, suggesting a link between overactivation of this pathway and induction of OCT4 and NANOG in residing pluripotent stem cells early during liver repair. In fact, β-Cat has been proposed as a novel regulator of NANOG to sustain liver pluripotency^48^. The implication of β-Cat signaling in AhR-dependent liver regeneration was also supported by additional observations: first, the marked increase in β-Cat levels shortly after damage in *AhR*−/− mice but not in *AhR*+/+ mice; second, *Axin2* was upregulated while *Dkk1* was repressed in basal *AhR*−/− livers, supporting the existence of an endogenous regulatory pathway; third, *Dkk1* was induced in AhR-null livers once regeneration reached its maximum, which suggested a mechanism to attenuate the process since Dkk1 can inhibit β-Cat signaling^57^. Thus, AhR and β-Cat may be interconnected in a regulatory network controlling the generation of stem-like and pluripotent cells needed for liver regeneration. This hypothesis is supported by the fact that AhR activation alters Wnt/β-Cat signaling impairing tissue regeneration in zebrafish^39, 58^ and the formation of the urogenital sinus during prostate development^59^.

In agreement with a previous study using a different AhR-null mice line^49^, AhR deficiency strongly increases the susceptibility to hepatocarcinogenesis eventually reducing survival in a significant fraction of mice. *AhR*−/− mice developed markedly large tumors, with increased multiplicity and vascularization and with complete penetrance. AhR expression affected malignancy since liver carcinomas could be recovered from *AhR*−/− mice whereas adenomas were predominant in *AhR*+/+ mice. Notably, the causal role of AhR in liver tumorigenesis gains additional support from the fact that, in *AhR*+/+ tumors, it was strongly repressed in the tumor proper but not in the surrounding non-tumoral parenchyma. Although more work is needed to clarify this differential pattern of expression, it agrees with our previous observations describing cell-autonomous and stromal dependent functions of AhR in melanoma primary tumorigenesis and metastasis^26^. Similarly to that found for CCl_4_-induced injury, β-Cat expression and activation were also increased in *AhR*−/− tumors, again suggesting the existence of a β-Cat/AhR signaling network limiting progression of liver carcinogenesis. Interestingly, OCT4 and NANOG expression did not significantly differ between *AhR*+/+ adenomas and *AhR*−/− carcinomas, a result that probably reflects a differential function of both genes in regeneration after acute toxicity with respect to liver carcinogenesis.

In summary, physiological control of AhR expression may be required to adjust a proportional proliferative response to repair necrotic damage in the liver caused by toxic chemicals. As AhR has relevant roles in controlling differentiation and pluripotency, and since β-Cat signaling promotes liver regeneration and is associated to the activation of pluripotency factors, it is reasonable to assume that a signaling network involving AhR and β-Cat could control the timing and the intensity of liver regeneration to restore tissue architecture and to avoid overgrowth. A similar network could also limit liver carcinogenesis in presence of AhR by restricting tumor growth and malignant progression. One relevant question that remains to be investigated is to what extent the effects reported here depend on the expansion of liver stem-like cells, on the absence of AhR in those cells or on both phenomena. The first possibility is supported by recent observations from our laboratory showing that AhR deficiency also improves lung regeneration after naphthalene-induced damage through an increase in the pool of basal and Clara stem-like cells (Hernández-Morales, unpublished observations), and that AhR-null testes have increased levels of early progenitor cells^25^. The second would imply that AhR deprivation triggers cell autonomous effects leading to undifferentiation and stemness. Indeed, AhR deficiency differentially affects melanoma primary tumorigenesis and metastasis depending on whether it is depleted in the tumor cell or in the stroma^26, 27^. Further, AhR knockdown enhances the undifferentiated phenotype of human embryonic teratocarcinoma cells by upregulating pluripotency genes OCT4 and NANOG^33^. It appears likely that AhR not only modulates stem cell function but also the expansion of undifferentiated cell populations. Further work will provide important insights into these novel roles of AhR.

## Methods

### Mice and treatments

*AhR*+/+ and *AhR*−/− mice were generated by gene targeting as previously described^35^. For liver regeneration experiments, male mice were used since the hormonal status of female rodents significantly affects the response to hepatotoxins^60–62^. Thus, four to five *AhR*+/+ and *AhR*−/− male mice for each time point at 12 to 14 weeks of age were treated with a single i.p. dose of 0.7 ml/Kg of pure CCl_4_ solution (Panreac 131245.1611) dissolved in sunflower oil; the same number of control mice received an equal volume of solvent. Mice were sacrificed 24 h, 48 h, 72 h, 4 days and 7 days after treatment and livers collected and processed for analysis. For liver tumor formation, 30 *AhR*+/+ and 30 *AhR*−/− male mice were injected twice at 4 and 5 weeks of age i.p. with 15 mg/Kg body weight diethylnitrosamine (DEN) (Sigma N0258) dissolved in sunflower oil. A set of 10 *AhR*+/+ and 10 *AhR*−/− control mice received the same volume of sunflower oil and they were followed during the duration of the experiment. All work involving mice has been performed in accordance with the National and European legislation (Spanish Royal Decree RD53/2013 and EU Directive 86/609/CEE as modified by 2003/65/CE, respectively) for the protection of animals used for research. Experimental protocols using mice were approved by the Bioethics Committee for Animal Experimentation of the University of Extremadura (Registry 109/2014) and by the Junta de Extremadura (EXP-20160506-1). Mice had free access to water and rodent chow.

### Antibodies and reagents

The following antibodies were used: anti-AhR (Immunostep EB11767 1:50), anti CK14 (COVANCE AF64 1:100), anti-OCT4 (Santa Cruz Biotechnology sc-5279, 1:200), anti-NANOG (Novus Biologicals NBP2-13177SS 1:50), anti-PCNA (Biolegend, 307902 1:50), anti-TBX3 (ABIN 1714884 1:50), anti-β-Cat (Becton-Dickinson 610153 1:200).

### Hematoxylin/eosin staining of liver sections

Liver tissues were fixed overnight at room temperature in buffered formalin and included in paraffin. Sections were prepared at 3 μm, deparaffinated in xylol and gradually re-hydrated to phosphate buffered saline (PBS). Tissues were incubated for 3 min with hematoxylin, washed with tap water and stained with eosin for 1 min. After a final washing step, sections were de-hydrated, mounted and observed in a NIKON TE2000U microscope equipped with 4× (0.10 numeric aperture), 10× (0.25 numeric aperture) and 20× (0.40 numeric aperture) objectives.

### Immunohistochemistry

Paraffin-embedded livers were sectioned at 3 μm, deparaffinated and re-hydrated to PBS. Sections were incubated for 45 min in PBS containing 0.25% Triton X-100 (PBS-T) and 0.3% H_2_O_2_ to block endogenous peroxidase activity. Following washing in PBS-T, unspecific binding was blocked by incubation in PBS-T solution containing 2 mg/ml gelatin (PBS-T-G) and 0.1 M lysine. A primary antibody against the proliferating cell nuclear antigen (PCNA) was added for 16–18 h and, after washing in PBS-T-G, sections were incubated for 1 h at room temperature with the corresponding biotin-conjugated secondary antibody. After a final washing step in PBS-T-G, the streptavidin-peroxidase complex was added and the presence of PCNA revealed with diaminobenzidine (DAB). Nuclei were then counterstained with Harris hematoxylin and sections de-hydrated and mounted with Eukitt (Kindler GmBH). A NIKON TE2000U microscope equipped with 4× (0.10 numeric aperture), 10× (0.25 numeric aperture) and 20× (0.40 numeric aperture) objectives was used for microscopic observation.

### Immunofluorescence

Liver sections (3 μm) were initially processed as indicated above but omitting the blocking step. Unspecific epitopes were blocked by 1 h incubation at room temperature in TBS-T containing 0.2% gelatin and 3% BSA. Sections were incubated overnight at 4 °C with the corresponding primary antibodies diluted in PBS-T containing 0.2% gelatin, washed in the same gelatin solution and incubated for 1 h at room temperature with Alexa-488 or Alexa-633- labeled secondary antibodies. After washing the excess of secondary antibody, sections were dehydrated, mounted on Mowiol and visualized using an Olympus FV1000 confocal microscope (Olympus). Objectives used were: 10× (0.40 numeric aperture) and 20× (0.70 numeric aperture). Fluorescence analysis was done using the FV10 software (Olympus). DAPI was used to stain cell nuclei.

### Reverse transcription and real-time PCR

Total RNA was purified from liver tissues at different times after CCl_4_ or DEN treatment. Tissues were grinded in liquid nitrogen, extracted using a Trizol reagent (Ambion)/ chloroform solution, centrifuged and the supernatants precipitated with isopropanol and centrifuged again at 15000 *g* for 30 min at 4 °C. Pellets were dissolved in DEPC-treated water and the resulting solution further purified with the High Pure RNA Isolation Kit (Roche). Reverse transcription was done using random priming and the iScript Reverse Transcription Super Mix (Bio-Rad). Real-time PCR (qPCR) was performed using SYBR® Select Master Mix (Life Technologies) in a Step One Thermal Cycler (Applied Biosystems) as indicated^25, 33^. *Gapdh* was used to normalize target gene expression (ΔCt) and 2^−ΔΔCt^ to calculate changes in mRNA levels with respect to untreated conditions. Primer sequences used are indicated in Supplementary Table 1.

### SDS-PAGE and immunoblotting

SDS-PAGE and immunoblotting were performed using total protein extracts essentially as described^25^. Briefly, *AhR*+/+ and *AhR*−/− liver tumors and surrounding non-tumoral tissue were minced, homogenized in ice-cold lysis buffer (50 mM Tris-HCl pH 7.5, 150 mM NaCl, 0.5% Nonidet P-40, 1 mM phenyl-methyl sulfonyl fluoride, 1 mM NaF, 1 mM sodium orthovanadate, 1 mM DTT, 10 mM β-glycerophosphate and 4 μg/μl Complete protease inhibitor cocktail) and centrifuged at 15000 *g* for 30 min at 4 °C. Protein concentration was quantified in the supernatants using the Coomassie Plus protein assay reagent (Pierce) and bovine serum albumin as standard. Aliquots of 20-30 μg total protein were electrophoresed in 8% SDS-PAGE gels which were transferred to nitrocellulose membranes by electroblotting. Membranes were blocked in TBS-T (50 mM Tris-HCl pH 7.5, 150 mM NaCl, 0.2% Tween-20) containing 5% non-fat milk. Blots were incubated with a primary antibody against AhR and with the corresponding secondary antibody, washed in TBS-T and revealed using the Super-signal luminol substrate (Pierce) in a ChemiDoc XRS + equipment (Bio-Rad).

### Side population (SP) isolation by cell sorting

The side population (SP) containing stem-like cells was isolated from *AhR*+/+ and *AhR*−/− livers essentially as described with some modifications^63^. In brief, livers were finely minced in PBS and rotated for 30 min at 37 °C in digestion solution (PBS containing 0.5 U/ml Dispase and 60 U/ml collagenase (Invitrogen)). Once digested, tissues were homogenized by passing through a 21-gauge syringe, filtered by a 0.40 μm mesh and centrifuged at 300 *g* for 5 min. Pellets were then resuspended in sorting medium (PBS containing 10% FBS) prior to use. To isolate SP cells, complete cellular pools obtained from each liver were stained for 90 min at 37 °C with a solution containing 5 μg/ml Hoechst 33342 (Sigma Aldrich). Cells were then centrifuged and resuspended in HEPES-HBSS buffer and incubated with 50 μM Fumitremorgin C (Sigma Aldrich) to inhibit the ABCG2 extrusion pump. Propidium iodide at a concentration of 10 nM was used to discriminate dead cells from the purification. Cells staining positive for the undifferentiation markers CD133 + , CD44 + and CD29 + were considered representative of the SP population. A MoFlo Astrium EQ flow cytometer (Beckman Coulter) was used.

### Statistical analyses

Quantitative data are shown as mean ± SD. Comparisons between experimental conditions was done using GraphPad Prism 6.0 software (GraphPad). The student´s t test was used to analyze differences between two experimental groups and ANOVA for the analyses of three or more groups. The Mann-Whitney non-parametric statistical method was used to compare rank variations between independent groups.

## Electronic supplementary material

Supplementary Table 1
