# Supplementary material for: Dioxin Receptor Adjusts Liver Regeneration After Acute Toxic Injury and Protects Against Liver Carcinogenesis
Source: Sci Rep. 2017 Sep 5;7:10420. doi: 10.1038/s41598-017-10984-w (PMC5585208; doi:10.1038/s41598-017-10984-w)
Supplement: Supplementary file 1 — Supplementary Table 1 [file 41598_2017_10984_MOESM1_ESM.pdf]

DIOXIN RECEPTOR ADJUSTS LIVER REGENERATION AFTER ACUTE TOXIC INJURY AND PROTECTS AGAINST LIVER CARCINOGENESIS

Nuria Moreno-Marín[1], Eva Barrasa[1], Antonio Morales-Hernández[2], Beroé Paniagua[1], Gerardo Blanco-Fernández[3], Jaime M. Merino[1] and Pedro M. Fernández-Salguero[1,*]

Supplementary Table 1

Oligonucleotide primers used in this study

Gene name	Direction	Primer sequence (5´-3´)
Axin 2	forward	ACTGGGTCGCTTCTCTTGAA
	reverse	CTCCCCACCTTGAATGAAGA
β-Cat	forward	CCCTGAGACCCTACATGAGG
	reverse	TGTCAGCTCAGGAATTGGAC
c-Jun	forward	TGAGTTGGCACCCACTGTTA
	reverse	TCCCCTATCGACATGGAGTC
Cyclin-D	forward	CACAACTTCTCGGCAGTCAA
	reverse	AGTGCGTGCAGAAGGAGATT
Dkk1	forward	GCAGGTGTGGAGCCTAGAAG
	reverse	GCCTCCGATCATCAGACTGT
Lef1	forward	GGGTGTTCTCTGGCCTTGT
	reverse	GCGACTTAGCCGACATCAA
Lrp6	forward	CTTTCTCGGGGTTTACCACA
	reverse	TGCAAACTCAGTCGCAAATC
Mmp2	forward	GCATTGGGTATCCATCCATC
	reverse	AGTCAGGGTCACCCACAAAG
Tcf4	forward	GTGACCCAAGATCCCTGCT
	reverse	CTTCTTTGGCGAGTGGACA
Cyp1a1	forward	ACAGACAGCCTCATTGAGCA
	reverse	GGCTCCACGAGATAGCAGTT
Gapdh	forward	TGAAGCAGGCATCTGAGGG
	reverse	CGAAGGTGGAAGAGTGGGAG
